# Long-Term Outcomes of Tuberous Sclerosis Complex-Associated Non-functional Pancreatic Neuroendocrine Tumors: Should We Be More Conservative?

**DOI:** 10.1245/s10434-023-14157-0

**Published:** 2023-08-30

**Authors:** Shahrzad Arya, Marco Ventin, Martina Nebbia, Carlos Fernandez-del Castillo, Gabriella Lionetto, Motaz Qadan, Keith D. Lillemoe, Vikram Deshpande, Onofrio A. Catalano, Elizabeth A. Thiele, Cristina R. Ferrone

**Affiliations:** 1 Department of Surgery, Massachusetts General Hospital, Harvard Medical School, Boston, MA USA; 2 Department of Pathology, Massachusetts General Hospital, Harvard Medical School, Boston, MA USA; 3 Department of Radiology, Massachusetts General Hospital, Harvard Medical School, Boston, MA USA; 4 Herscot Center for Tuberous Sclerosis Complex, Massachusetts General Hospital, Harvard Medical School, Boston, MA USA; 5 Department of Surgery, Cedars-Sinai Medical Center, Los Angeles, CA USA

**Keywords:** Pancreatic neuroendocrine tumor, PNET, Tuberous sclerosis complex syndrome, TSC, Outcomes, mTOR inhibitor, Pancreatic lesion

## Abstract

**Background:**

Hereditary syndromes such as tuberous sclerosis complex (TSC) account for 10% of pancreatic neuroendocrine tumors (PNETs). Surgical intervention is the current standard of care for sporadic PNETs (spPNETs) that are >2 cm in size. We compared the long-term outcomes of resected TSC-PNETs with patients with spPNETs.

**Methods:**

We conducted a retrospective review of perioperative data and outcomes of TSC-PNETs compared with spPNETs. Inclusion criteria involved selecting patients whose tumors were no larger than 5.1 cm, the maximum size observed in the TSC-PNET group.

**Results:**

Of the 347 patients resected for PNETs, 14 were TSC-PNETs and 241 were non-functional spPNETs. The median age for the whole cohort was 56 years (interquartile range [IQR] 21.0) and 47% were female. The median follow-up was 103.8 months (95% confidence interval [CI] 89.2–118.6). Specifically, 14 patients with TSC-PNETs and 194 patients with spPNETs were included. Compared with spPNETs, patients with TSC-PNETs were operated on at a younger age (24.0 vs. 57.5 years; *p* < 0.001), were more frequently multifocal (28.5% vs. 0.0%; *p* < 0.001), were more likely to undergo minimally invasive operations (78.6% vs. 24.3%; *p* < 0.001), and had more R1 resections (28.6% vs. 5.7%; *p* = 0.006). Local and distant tumor recurrence was only observed in the spPNET group. The 5-year mortality rates for the spPNET and TSC-PNET groups were 6.2% and 0.0%, respectively. No PNET-related deaths were observed among TSC-PNETs.

**Conclusion:**

None of the TSC-PNET patients recurred after a median follow-up of 78.0 months. The risk-benefit of aggressive pancreatic operations in TSC-PNET patients is still unclear and our findings suggest a conservative approach should be considered.

Tuberous sclerosis complex (TSC) is a rare autosomal dominant genetic syndrome with an approximate incidence of 1 in 5000–10,000 live births.^1,2^ TSC patients have a mutation in the TSC1 or TSC2 genes, which encode for hamartin and tuberin, respectively.^1,3^ These inactivating mutations result in the deregulation of the mammalian target to rapamycin (mTOR) pathway. This alteration of the mTOR pathway results in dysregulation of cell growth and metabolism,^4^ which is why TSC patients develop benign tumors in multiple organ systems, including the brain, kidneys, lungs, heart, eyes, and skin.^1,3^ An association between TSC and pancreatic neuroendocrine tumors (PNETs) has recently been established.^4–8^

PNETs are a rare and heterogeneous neoplasm with an incidence of five cases per million.^9,10^ In contrast, the prevalence rate in autopsy cases has been reported to be as high as 10%.^11^ Although most PNETs occur sporadically, about 10% are due to hereditary syndromes, including multiple endocrine neoplasia type 1 (MEN1), multiple endocrine neoplasia type 4 (MEN4), von Hippel–Lindau disease (VHL), neurofibromatosis type 1 (NF1), and TSC.^12^

The clinical course of PNETs in patients with MEN1, VHL, and NF1 syndromes tends to be more favorable when compared with sporadic PNETs (spPNETs).^7,10^ An operation is currently the standard of care for all well-differentiated grade 1 and 2 PNETs >2 cm, a growth rate >0.5 cm in 6–12 months, grade 3 tumors, and functional or symptomatic PNETs. To date, disease-specific guideline recommendations are missing for TSC-associated PNETs (TSC-PNETs).^13,14^ However, clinical decisions about the management of PNETs diagnosed in TSC patients, who generally have a high number of clinically relevant comorbidities, are often challenging due to the morbidity and mortality associated with pancreatic operations.^14^

A comparative analysis of long-term outcomes between spPNETs and TSC-PNETs is lacking. The current study evaluates the clinical course of patients who have undergone surgical resection of their PNETs, specifically comparing the disease course of TSC-PNETs with patients with nonfunctional spPNETs.

## Methods

### Study Design and Population

This single-center, retrospective, comparative study of TSC-PNET and spPNET patients was approved by the Institutional Review Board. Patients undergoing pancreatic resection for PNETs at the Massachusetts General Hospital (MGH) between December 1998 and December 2020 were evaluated, and patients with TSC-PNETs were compared with patients with non-functional PNETs. Patients with functional tumors or other hereditary syndromes were excluded from this study.

Clinicopathologic factors including sex, age at operation, body mass index (BMI), American Society of Anesthesiologists (ASA) score, and type of operation (pancreatoduodenectomy, distal pancreatectomy, total pancreatectomy, central pancreatectomy, or pancreatic enucleation) were evaluated.

Histopathological parameters evaluated were tumor size, tumor location (pancreatic head, body or tail, and multifocal tumor), lesion type (solid, cystic, and mixed solid/cystic), TNM stage (American Joint Committee on Cancer [AJCC] 8th edition), Ki-67 proliferation index, tumor grading according to the 2010 World Health Organization (WHO) classification, number of harvested lymph nodes, and number of positive lymph nodes. Negative resection margin (R0) status was defined according to the Union for International Cancer Control (UICC)/AJCC if no tumor cells were detected within 1 mm of the resection margin, and R2 status was defined as macroscopic evidence of a tumor left in situ. Postoperative complications were determined according to the Clavien–Dindo Classification and dichotomized. Local or distant tumor recurrences were classified based on pathology or imaging. Disease-free survival (DFS) was defined as the time between the operation and the first tumor recurrence, while overall survival (OS) time was defined from the date of operation until death of any cause.

### Statistical Analysis

Continuous variables were expressed as medians and interquartile range (IQR), and categorical variables were expressed as absolute numbers and relative frequencies. A case-control matching was performed between patients with TSC-PNETs and spPNETs based on the pathological size of the largest tumor. Group analyses of categorical variables were performed using the Chi-square test or Fisher’s exact test with Bonferroni correction. Continuous variables were compared among groups, using the non-parametric Kruskal–Wallis test, and the median follow-up period was calculated using the reverse Kaplan–Meier method. Survival analyses were performed using the Kaplan–Meier method with log-rank test, and statistical analysis was performed using IBM SPSS Statistics (version 28.0; IBM Corporation, Armonk, NY, USA).

## Results

### Patient Characteristics

A total of 347 patients underwent pancreatectomy for PNETs. After excluding patients with functional and other hereditary syndrome-associated PNETs, 255 patients were considered eligible for this study; 14 were TSC-PNETs and 241 were non-functional spPNETs. Patient and operative characteristics are shown in Table 1. Males constituted 52.9% (*n* = 135) of the cohort, and the median age at operation was 56.0 years (IQR 21.0). TSC patients were operated on at a younger age (24 years vs. 57 years; *p* < 0.001) and were more likely to undergo minimally invasive operations compared with spPNETs (78.6% vs. 21.6%; *p* < 0.001).

**Table 1:** Clinicopathological patient characteristics and outcomes of the entire cohort

Variable	Overall [*n* = 255]	spPNET [*n* = 241]	TSC-PNET [*n* = 14]	*p*-Value
Male sex	135 (52.9)	128 (53.1)	7 (50.0)	0.821
Age at operation, years [median (IQR)]	56.0 (21.0)	57.0 (20.0)	24.0 (22.0)	**< 0.001**
Minimally invasive surgery	63 (24.7)	52 (21.6)	11 (78.6)	**< 0.001**
Parenchyma-sparing surgery	46 (18.0)	45 (18.7)	1 (7.14)	0.244
Tumor size, pathology [median (IQR)]	2.7 (2.5)	2.7 (2.5)	2.7 (2.8)	0.886
Lesion type				0.324
Solid	191 (76.2)	183 (76.9)	8 (57.2)	
Cystic	36 (13.9)	33 (13.9)	3 (21.4)	
Mixed	25 (9.9)	22 (9.2)	3 (21.4)	
Tumor location				**< 0.001**
Pancreatic head	76 (29.8)	75 (31.1)	1 (7.1)	
Pancreatic body or tail	175 (68.6)	166 (68.9)	9 (64.3)	
Multifocal	4 (1.6)	0 (0.0)	4 (28.6)	
WHO grade				0.691
I	154 (62.9)	144 (62.3)	10 (71.4)	
II	84 (34.3)	80 (34.6)	4 (28.6)	
III	7 (2.9)	7 (3.0)	0 (0.0)	
Ki-67 index				0.670
>3	81 (31.8)	78 (32.4)	3 (21.4)	
Resection margin [R status]				**0.004**
R0	223 (87.8)	215 (89.2)	9 (64.3)	
R1	19 (7.5)	15 (6.2)	4 (28.6)	
R2	12 (4.7)	11 (4.6)	1 (7.1)	
T				0.764
T1-T2	180 (70.6)	169 (70.1)	11 (78.6)	
T3	75 (29.4)	72 (29.9)	3 (21.4)	
N				**0.027**
N1	61 (23.9)	61 (25.3)	0 (0.0)	
Not determined	52 (20.4)	46 (19.1)	6 (42.9)	
M				0.215
M0	231 (90.6)	217 (90.0)	14 (100.0)	
M1	24 (9.4)	24 (10.0)	0 (0.0)	
Vascular invasion	77	75	2 (14.3)	0.158
Perineural invasion	56	55	1 (7.1)	0.138
Harvested lymph nodes [median (IQR)]	5.0 (13.0)	6.0 (14.0)	1.5 (3.0)	**0.005**
Positive lymph nodes [median (IQR)]	0.0 (1.0)	0.0 (1.0)	0.0 (0.0)	0.064
Lymph node ratio [median (IQR)]	0.0 (0.1)	0.0 (0.1)	0.0 (0.0)	0.065
Postoperative complications				0.262
Clavien-Dindo ≥3	47 (18.4)	46 (19.1)	1 (7.1)	
Length of stay [median (IQR)]	6.0 (3.0)	6.0 (2.0)	5.0 (4.0)	0.155
Tumor recurrence	44 (17.3)	44 (18.3)	0 (0.0)	0.081
Recurrence location				0.378
Local	8 (3.1)	8 (3.3)	0 (0.0)	
Distant	30 (11.8)	30 (12.4)	0 (0.0)	
Local + distant	6 (2.4)	6 (2.5)	0 (0.0)	
5-year disease-specific survival	15 (5.9)	15 (6.2)	0 (0.0)	0.336
10-year disease-specific survival	27 (10.6)	27 (11.2)	0 (0.0)	0.185

Bold values indicate the statistical significance at an alpha level of 0.05

Data are expressed as *n* (%) unless otherwise specified

*spPNETs* sporadic PNETs, *TSC* tuberous sclerosis complex, *PNETs* pancreatic neuroendocrine tumors, *IQR* interquartile range, *WHO* World Health Organization

### Tumor Characteristics and Prognosis

**Fig. 1:**
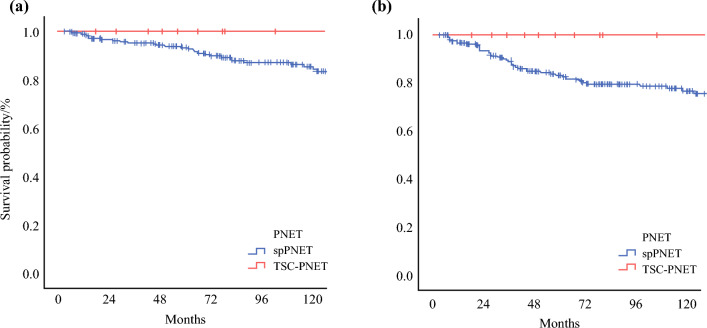
Kaplan–Meier survival curves showing (a) overall survival and (b) disease-free survival of spPNETs and TSC-PNETs. *spPNETs* sporadic PNETs, *TSC* tuberous sclerosis complex, *PNETs* pancreatic neuroendocrine tumors

Tumor characteristics and distribution of prognostic factors between spPNETs and TSC-PNETs are presented in Table 1. The median tumor size for the whole cohort was 2.7 cm (IQR 2.5). Final pathology revealed a well-differentiated (WHO grade I) tumor in most (62.9%) patients, and there were no significant differences between the TSC-PNET and spPNET cohorts (*p* = 0.7). Cases of poorly differentiated (grade III) tumors were rare and were only observed in patients with spPNETs (*n* = 7, 3.0%). TSC-PNETs were more frequently multifocal (28.5% vs. 0.0%; *p* < 0.001). Lymph node (N1) and distant metastases (M1) were only observed among patients with spPNETs (25.3% vs. 0.0%, *p* = 0.027; and 10.0% vs. 0.0%, *p* = 0.215) respectively). The median follow-up period of the entire cohort was 103.8 months (95% confidence interval [CI] 89.2–118.6), and the median follow-up period for TSC-PNET and spPNET patients was 78.0 (95% CI 52.2–103.9) and 106.7 (95% CI 89.3–124.0) months, respectively. Despite a higher rate of R1 resections (28.6% vs. 6.2%; *p* = 0.004), TSC-PNETs did not recur locally or distantly. Local (*n* = 8, 3.3%), distant (*n* = 30, 12.4%), and local + distant (*n* = 6, 2.5%) recurrence were only observed among spPNETs. Median OS and DFS were not reached in both cohorts. Kaplan–Meier survival curves are shown in Fig. 1. In 14 resected TSC-PNETs, three patients developed new-onset diabetes, and one patient who underwent subtotal pancreatectomy was taking pancreatic enzyme supplements (Table 2).

**Table 2:** Case-control-matched representation of spPNET and TSC-PNET patient demographic characteristics, clinicopathological data, and outcomes

Variable	Overall [*n* = 208]	spPNET [*n* = 194]	TSC-PNET [*n* = 14]	*p*-Value
Sex				0.765
Female	96 (46.2)	89 (45.9)	7 (50.0)	
Male	112 (53.8)	105 (54.1)	7 (50.0)	
Age at operation, years [median (IQR)]	56.0 (21.0)	57.5 (19.0)	24.0 (22.0)	**<0.001**
Minimally invasive surgery	58 (27.9)	47 (24.3)	11 (78.6)	**<0.001**
Parenchyma-sparing surgery	42 (20.2)	41 (21.1)	1 (7.1)	0.31
Tumor size (pathology), cm [median (IQR)]	2.4 (1.7)	2.4 (1.7)	2.7 (2.8)	0.422
Lesion type				0.134
Solid	154 (74.8)	146 (76.0)	8 (57.2)	
Cystic	33 (16.0)	30 (15.6)	3 (21.4)	
Mixed	19 (9.2)	16 (8.3)	3 (21.4)	
Tumor location				**<0.001**
Pancreatic head	66 (31.7)	65 (33.5)	1 (7.1)	
Pancreatic body or tail	138 (66.3)	129 (66.5)	9 (64.3)	
Multifocal	4 (1.9)	0 (0.0)^a^	4 (28.5)^b^	
WHO grade, 2010				0.795
I	140 (69.0)	130 (68.8)	10 (71.4)	
II	57 (28.1)	53 (28.0)	4 (28.6)	
III	6 (3.0)	6 (3.2)	0 (0.0)	
Ki-67 index				0.629
>3	57 (27.4)	54 (27.8)	3 (21.4)	
Resection margin [R status]				**0.006**
R0	185 (89.4)	177 (91.2)	9 (64.3)	
R1	15 (7.2)	11 (5.7)^a^	4 (28.6)^b^	
R2	7 (3.4)	6 (3.1)	1 (7.1)	
T				0.410
T1-T2	180 (86.5)	169 (87.1)	11 (78.6)	
T3	28 (13.5)	25 (12.9)	3 (21.4)	
N				**0.023**
N0	117 (56.3)	109 (56.2)	8 (57.1 )	
N1	41 (19.7)	41 (21.1)^a^	0 (0.0)^b^	
Not determined	50 (24.0)	44 (22.7)	6 (42.9)	
M				0.607
M0	192 (92.3)	178 (91.8)	14 (100.0)	
M1	16 (7.7)	16 (8.2)	0 (0.0)	
Vascular invasion	54 (28.7)	52 (29.7)	2 (14.3)	0.354
Perineural invasion	43 (23.6)	42 (24.9)	1 (7.1)	0.306
No. of harvested lymph nodes [median (IQR)]	5.0 (13.0)	5.0 (13.0)	1.5 (3.0)	**0.016**
No. of positive lymph nodes [median (IQR)]	0.0 (1.0)	0.0 (1.0)	0.0 (0.0)	0.091
Lymph node ratio [median (IQR)]	0.0 (0.04)	0.0 (0.05)	0.0 (0.0)	0.091
Postoperative complications				
Clavien–Dindo ≥3	40 (19.2)	39 (20.1)	1 (7.1)	0.314
Length of stay [median (IQR)]	6.0 (2.0)	6.0 (2.0)	5.0 (4.0)	0.121
Tumor recurrence	30 (14.9)	30 (16.0)	0 (0.0)	0.233
Recurrence location				0.668
Local	5 (2.4)	5 (2.6)	0 (0.0)	
Distant	21 (10.1)	21 (10.8)	0 (0.0)	
Local + distant	4 (1.9)	4 (2.1)	0 (0.0)	
5-year disease-specific mortality	12 (5.8)	12 (6.2)	0 (0.0)	0.338
10-year disease-specific mortality	22 (10.6)	22 (11.3)	0 (0.0)	0.183

Bold values indicate the statistical significance at an alpha level of 0.05

Data are expressed as *n* (%) unless otherwise specified

*spPNETs* sporadic PNETs, *TSC* tuberous sclerosis complex, *PNETs* pancreatic neuroendocrine tumors, *IQR* interquartile range, *WHO* World Health Organization

^a,b^Statistically significant difference between column proportions

### Case-Control Matching

Case-control matching based on the largest tumor size was performed for 14 patients with TSC-PNETs and 194 patients with spPNETs. All PNETs were <5.1 cm in size, which was the maximum size observed in the TSC-PNET cohort. TSC-PNETs were operated on at a younger age (24.0 years vs. 57.5 years; *p* < 0.001) and were more likely to undergo minimally invasive operations compared with spPNETs (78.6% vs. 24.3%; *p* < 0.001). There were no significant differences in tumor type (solid vs. cystic; *p* = 0.13) and tumor WHO grade (*p* = 0.795). The number of harvested lymph nodes was higher in the spPNET group (5.0 [IQR 13.0] vs. 1.5 [IQR 3.0]; *p* = 0.016). Lymph node metastases were only observed in the spPNET group (21.1% vs. 0.0%; *p* = 0.023), while postoperative complications (*p* = 0.31) and length of hospital stay (*p* = 0.12) were comparable between the two groups.

Compared with spPNETs, TSC-PNETs were more frequently multifocal (28.5% vs. 0.0%; *p* < 0.001). Among the multifocal TSC-PNETs, most patients had tumors in the body/tail of the pancreas, with an average of two to three tumors per patient. Distal/subtotal pancreatectomy, with or without enucleation, was successful in removing these multifocal tumors in three of four patients with tumor-free resection margins. Pathological analysis of all multifocal tumors demonstrated that they were grade 1, with a ki67% proliferation index below 3%. In a patient with multifocal TSC-PNETs involving the head, uncinate process, and tail of the pancreas, two lesions in the head and uncinate process were left in situ during the operation since a total pancreatectomy was judged not suitable for the patient. The tumor removed in the tail was 2.2 cm, and all lymph nodes were negative. The background pancreas was remarkable for multiple neuroendocrine microadenomas ranging in size from 0.2 to 0.35 cm in the greatest dimension. The remaining PNETs measured 2.1 cm and 1.6 cm and have not progressed over 23.8 months.

Among spPNETs, tumor recurrence was observed in 30 (16.0%) patients, of whom 20 patients presented with isolated liver metastases, 4 had a local and liver recurrence, and 5 had local recurrence only. One patient developed recurrent multiorgan disease involving the liver, peritoneum, and bone.

Only one TSC-PNET patient presented with a new 8 mm T2-weighted hyperintense non-enhancing pancreatic lesion adjacent to the resection margin on magnetic resonance imaging (MRI) 33.8 months after the R0 resection of a well-differentiated PNET in the pancreatic tail. The 5- and 10-year mortality rates of spPNET and TSC-PNET patients were 6.2% vs. 0.0% and 11.3% vs. 0.0%, respectively. No PNET-related deaths were observed among the TSC-PNET patients. OS and DFS were not reached.

Given the fact that none of the TSC patients had nodal metastases or poorly differentiated grade 3 histology, we further compared outcomes of patients with spPNETs, matched for tumor size and absence of lymph node metastases and grade 3 histology, with the TSC group. After exclusion of patients (*n* = 47) with the latter risk factors, a lower overall tumor recurrence rate of 8.5% was observed. However, tumor recurrence from spPNETs was still significantly higher compared with TSC-PNETs (9.3% vs. 0.0%; *p* = 0.035). Similar findings applied for 5- (4.0%) and 10-year (9.3%) mortality rates from spPNETs, which slightly improved in the case of grade 1–2 tumors and lymph node-negative disease.

## Discussion

PNETs account for 1–2% of all pancreatic tumors, with TSC patients at higher risk of developing PNETs than the general population. This is the first study to compare the postoperative outcomes of TSC-PNET patients with spPNET patients. Currently, TSC-PNET patients are managed similarly to spPNET patients, however there are insufficient data to support appropriate decision making in this rare cohort.^13–16^ This study provides further data on the natural history of TSC patients who develop PNETs.

As imaging has improved over the years, more PNETs are being identified.^6,7,17–34^ TSC patients undergo a routine abdominal MRI every 1–3 years to assess the commonly associated renal angiolipomas. With an improved understanding of TSC-associated PNETs, we would recommend adding pancreatic imaging during the abdominal MRI.^7,16^

TSC-PNET patients may have a more benign disease course and a better outcome compared with spPNETs. In the literature, 56 TSC patients developed a PNET,^6,17–35^ of whom only one PNET-related death was reported due to systemic metastases.^36^ Additionally, of these 56 patients, only 5 had peripancreatic lymph node metastases.^17–19^ In our cohort of spPNET patients, 25.3% (61/241) had lymph node metastases and 18.3% (44/241) developed tumor recurrence, which is in contrast to the 14 TSC-PNET patients who have not recurred after a median follow-up of 78 months.

After case-control matching based on PNET size, lymph node metastases and tumor recurrence occurred in 21.1% (41/194) and 16.0% (30/194) of spPNET patients. Interestingly, after a median follow-up of 78 months, the TSC-PNET patients did not present with local or distant recurrence, even in the setting of an R1 resection, vascular invasion, or perineural invasion, traditionally considered negative prognostic factors.^10,13,14^ These findings suggest that TSC-PNETs may have a more indolent biology and disease course. Compared with spPNETs, TSC-PNETs present at a younger age and demonstrate better long-term outcomes. Although the reason for better outcomes in TSC-PNET patients is still unclear, conservative strategies such as regular follow-up imaging could be considered due to the benign nature of their PNETs.^6,20^ For patients who may require an operation, parenchyma-sparing resections rather than formal resections should be considered to preserve pancreatic parenchyma and decrease the operative complication rates.^37^

This favorable long-term outcome is also documented in other hereditary syndrome-associated PNETs, including MEN1, VHL, and NF1.^12,38^ Hereditary syndrome-associated PNETs, especially those associated with MEN1 syndrome, are more frequently multifocal compared with spPNETs.^12^ Interestingly, most of these MEN1-associated multifocal PNETs are originally independent tumors or the combination of independent tumors with intrapancreatic tumor expansion.^39^ Sporadic or syndromic multifocal PNETs have not been associated with a worse prognosis.^8,40^ Similarly, in our study, four TSC patients presented with multifocal PNETs without evidence of worse outcomes. The favorable long-term prognosis of resected PNETs in patients with VHL is also not influenced by small tumors left in the pancreas.^38^ The similar findings observed in our cohort may generate further evidence supporting the benign nature of PNETs occurring in TSC patients. In 14 resected TSC-PNETs, three patients developed new-onset diabetes. Therefore, unless a rapid interval increase in size is observed, the mild behavior of these lesions suggests that clinical observation can be a viable option.

Conservative strategies, including clinical monitoring and medical treatment with mTOR inhibitors, have recently been suggested as a potential alternative to an operation in TSC patients with PNETs. The mTOR signaling pathway plays a significant role in cell growth and proliferation and contributes to the development of tumors in TSC patients due to the loss-of-function mutations involving the TSC1/TSC2 genes. Of note, mTOR inhibitors have been US FDA-approved for the treatment of several TSC-associated lesions, such as subependymal giant cell astrocytoma, kidney angiolipoma, lung lymphangioleiomyomatosis, and skin angiofibroma.^16^

Interestingly, among TSC patients receiving systemic mTOR inhibitors for the treatment of other TSC-related tumors, modest beneficial effects have been observed in decreasing the growth rate of concomitant PNETs or pathologically undefined pancreatic lesions.^6,20^ Similarly, one of our TSC patients with a biopsy-proven PNET received an mTOR inhibitor (sirolimus) for renal angiomyolipomas and a subependymal giant cell tumor of the brain. This patient experienced a 15% decrease in the size of the pancreatic lesion over 4.4 years.^5^ The predominant role of the mTOR pathway in the tumorigenesis and progression of neuroendocrine tumors (NETs) has previously been demonstrated in preclinical studies and clinical trials investigating the role of mTOR inhibitors for the treatment of metastatic NETs.^4^ Overall, these findings seem to suggest there might be a potential role for mTOR inhibitors in the therapeutic algorithm of TSC patients with PNETs; however, it is not clear whether mTOR inhibitors can be used as a substitute for an operation. Unfortunately, due to the rarity of TSC, it is unlikely that this question will be answered in a clinical trial.

This study is limited due to its retrospective design, which could potentially introduce selection and information bias. The study also had a small sample size, which leads to less statistically powerful 1:1 matching.

## Conclusion

Overall, TSC-PNETs appear to have a benign nature, and local/distant metastases as well as tumor recurrence after an operation are very rare. Compared with spPNET patients, TSC-PNETs present at a younger age and demonstrate favorable postoperative long-term outcomes. Further adoption of mTOR inhibitors in complex TSC patients with concomitant PNETs, and their close observation, could contribute to informatively guiding clinical decisions and building references for the management of PNETs in TSC patients. The risk-benefit of an aggressive pancreatic operation in TSC-PNET patients is still unclear and our findings suggest a conservative approach should be considered.

## References

[1] Curatolo P, Bombardieri R, Jozwiak S (2008). Tuberous sclerosis. Lancet..

[2] Hong CH (2016). An estimation of the incidence of tuberous sclerosis complex in a nationwide retrospective cohort study (1997–2010). Br J Dermatol..

[3] Henske EP (2016). Tuberous sclerosis complex. Nat Rev Dis Primers..

[4] Lamberti G (2018). The role of mTOR in neuroendocrine tumors: future cornerstone of a winning strategy?. Int J Mol Sci..

[5] Evans LM (2022). Tuberous sclerosis complex-associated nonfunctional pancreatic neuroendocrine tumors: Management and surgical outcomes. Am J Med Genet A..

[6] Mowrey K (2021). Frequency, progression, and current management: report of 16 new cases of Nonfunctional pancreatic neuroendocrine tumors in tuberous sclerosis complex and comparison with previous reports. Front Neurol..

[7] Larson AM (2012). Pancreatic neuroendocrine tumors in patients with tuberous sclerosis complex. Clin Genet..

[8] Sauter M (2021). Rare manifestations and malignancies in tuberous sclerosis complex: findings from the TuberOus SClerosis registry to increAse disease awareness (TOSCA). Orphanet J Rare Dis..

[9] Das S, Dasari A (2021). Epidemiology, incidence, and prevalence of neuroendocrine neoplasms: are there global differences?. Curr Oncol Rep..

[10] Halfdanarson TR (2008). Pancreatic endocrine neoplasms: epidemiology and prognosis of pancreatic endocrine tumors. Endocr Relat Cancer..

[11] Kimura W, Kuroda A, Morioka Y (1991). Clinical pathology of endocrine tumors of the pancreas Analysis of autopsy cases. Dig Dis Sci..

[12] Soczomski P (2021). A direct comparison of patients with hereditary and sporadic pancreatic neuroendocrine Tumors: evaluation of clinical course, prognostic factors and genotype-phenotype correlations. Front Endocrinol Lausanne.

[13] Landoni L (2019). The evolution of surgical strategies for pancreatic neuroendocrine tumors (Pan-NENs): time-trend and outcome analysis from 587 consecutive resections at a high-volume institution. Ann Surg..

[14] Falconi M (2016). ENETS consensus guidelines update for the management of patients with functional pancreatic neuroendocrine tumors and non-functional pancreatic neuroendocrine tumors. Neuroendocrinology..

[15] Zhang IY (2016). Operative versus nonoperative management of nonfunctioning pancreatic neuroendocrine tumors. J Gastrointest Surg..

[16] Northrup H (2021). Updated international tuberous sclerosis complex diagnostic criteria and surveillance and management recommendations. Pediatr Neurol..

[17] Verhoef S (1999). Malignant pancreatic tumour within the spectrum of tuberous sclerosis complex in childhood. Eur J Pediatr..

[18] Arva NC (2012). Well-differentiated pancreatic neuroendocrine carcinoma in tuberous sclerosis–case report and review of the literature. Am J Surg Pathol..

[19] Díaz Díaz D (2012). Neuroendocrine tumor of the pancreas in a patient with tuberous sclerosis: a case report and review of the literature. Int J Surg Pathol..

[20] Koc G (2017). Pancreatic tumors in children and young adults with tuberous sclerosis complex. Pediatr Radiol..

[21] Al Qahtani MS (2021). Insulinoma in tuberous sclerosis: An entity not to be missed. Saudi Med J..

[22] Amarjothi J (2019). Interesting pancreatic tumour in the background of Tuberous Sclerosis. BMJ Case Rep..

[23] Mehta S (2019). Pancreatic neuroendocrine tumor in a young child with tuberous sclerosis complex 1. J Endocr Soc..

[24] Mortaji P (2018). Pancreatic neuroendocrine tumor in a patient with a TSC1 variant: case report and review of the literature. Fam Cancer..

[25] Bombardieri R (2013). Pancreatic neuroendocrine tumor in a child with a tuberous sclerosis complex 2 (TSC2) mutation. Endocr Pract..

[26] Sreenarasimhaiah J (2009). Pancreatic somatostatinoma and tuberous sclerosis: case report of an exceedingly rare association. Gastrointest Endosc..

[27] Merritt JL (2006). Extensive acrochordons and pancreatic islet-cell tumors in tuberous sclerosis associated with TSC2 mutations. Am J Med Genet A..

[28] Francalanci P (2003). Malignant pancreatic endocrine tumor in a child with tuberous sclerosis. Am J Surg Pathol..

[29] Eledrisi MS, Stuart CA, Alshanti M (2022). Insulinoma in a patient with tuberous sclerosis: is there an association?. Endocr Pract..

[30] Kim H, Kerr A, Morehouse H (1995). The association between tuberous sclerosis and insulinoma. AJNR Am J Neuroradiol..

[31] Schwarzkopf G, Pfisterer J (1994). Metastasizing gastrinoma and tuberous sclerosis complex Association or coincidence in German. Zentralbl Pathol..

[32] Davoren PM, Epstein MT (1992). Insulinoma complicating tuberous sclerosis. J Neurol Neurosurg Psychiatry..

[33] Ilgren EB, Westmoreland D (1984). Tuberous sclerosis: unusual associations in four cases. J Clin Pathol..

[34] Gutman A, Leffkowitz M (1959). Tuberous sclerosis associated with spontaneous hypoglycaemia. Br Med J..

[35] van den Akker M (2012). Malignant pancreatic tumors in children: a single-institution series. J Pediatr Surg..

[36] Amin S (2017). Causes of mortality in individuals with tuberous sclerosis complex. Dev Med Child Neurol..

[37] Bolm L (2022). Long-term outcomes of parenchyma-sparing and oncologic resections in patients with nonfunctional pancreatic neuroendocrine Tumors <3 cm in a large multicenter cohort. Ann Surg..

[38] de Mestier L (2015). Long-term prognosis of resected pancreatic neuroendocrine tumors in von hippel-lindau disease is favorable and not influenced by small tumors left in place. Ann Surg..

[39] Katona TM (2006). Molecular evidence for independent origin of multifocal neuroendocrine tumors of the enteropancreatic axis. Cancer Res..

[40] Gudmundsdottir H (2021). Multifocality is not associated with worse survival in sporadic pancreatic neuroendocrine tumors. J Surg Oncol..

